# Editorial: Neuroimaging of Cognitive and Neuropsychiatric Symptoms in Movement Disorders

**DOI:** 10.3389/fneur.2022.843440

**Published:** 2022-01-31

**Authors:** Frederic Sampedro, Estela Camara, Jaime Kulisevsky

**Affiliations:** ^1^Movement Disorders Unit, Neurology Department, Hospital de la Santa Creu i Sant Pau, Barcelona, Spain; ^2^Biomedical Research Institute (IIB-Sant Pau), Barcelona, Spain; ^3^Centro de Investigación en Red-Enfermedades Neurodegenerativas (CIBERNED), Barcelona, Spain; ^4^Cognition and Brain Plasticity Unit, Institut d'Investigació Biomèdica de Bellvitge (IDIBELL), Barcelona, Spain; ^5^Cognition, Development and Educational Psychology, University of Barcelona, Barcelona, Spain

**Keywords:** neuroimaging, movement disorders, cognitive symptoms, neuropsychiatric symptoms, genetic modifiers

Movement disorders are relatively frequent neurological disorders that can have a profound impact on the patients' quality of life. Examples of movement disorders are Parkinson's disease (PD) and Huntington's disease (HD). Even though they are best known for its cardinal motor symptoms, most of the patients show concomitant cognitive or neuropsychiatric disturbances, which can be as debilitating as motor symptoms and have, unfortunately very limited therapeutic options.

The pathological hallmarks of PD and HD are, respectively, subcortical dopaminergic depletion due to substantia nigra degeneration and caudate atrophy due to aggregation of mutant huntingtin. These are responsible for the characteristic motor symptoms observed in the patients. Conversely, the origin of non-motor symptoms in PD or HD is not fully understood. For many years, it was assumed that they were a mere consequence of the inherent subcortical damage occurring in these disorders.

However, it is now clear that early cortical—and not only subcortical—damage occurs in these disorders, playing in turn a key role in the development and severity of cognitive and behavioral disturbances. Nonetheless, the neuropathological processes leading to cortical degeneration in movement disorders remain elusive, and are likely to differ from those leading to subcortical damage. Therefore, a precise understanding of these processes and its association with non-motor symptoms is urgently needed to design optimal therapeutic strategies.

In this context, neuroimaging techniques have played a key role in unraveling not only the presence of cortical damage and its association with non-motor symptoms, but also contributing to our understanding of its pathological origin. This Research Topic succeeded at providing high quality contributions in this research field. Here, a brief introduction to the 11 accepted papers is given. We refer the readers to the papers in this topic and the references therein for more details.

Martín-Bastida et al. provided an excellent review of the imaging alterations underlying cognitive impairment and impulse control disorders in PD, highlighting its potential use as diagnostic, prognostic or monitoring indicators.

In PD, beta-amyloid aggregation has been suggested to be one of the possible pathological entities contributing to cortical damage and cognitive decline. However, whether amyloid pathology is an inherent process in PD or rather reflects concurrent co-morbid Alzheimer's disease (AD) pathology, has not been fully elucidated. In fact, contradictory results have been published in this context. The fact that Melzer et al. were unable to find an association between amyloid PET and cognitive performance in a PD sample suggest that, at least, that amyloid pathology would not be the primary driver of cognitive impairment and dementia in most PD patients. This hypothesis was further reinforced by the findings of Antonini et al.

Genetic risk factors such as the MAPT H1H1 haplotype have also been related to cognitive impairment in PD, and Sampedro et al. showed that this genetic variant is also associated with cortical gray matter loss. It should not be overlooked that age is a constant and strong risk factor cognitive disturbances in PD. The specific mechanisms involved in this association were assessed by Nagano-Saito et al. using resting-state fMRI data, unraveling the a pattern of hub alterations and compensatory mechanisms associated with age in PD. Resting-state data also revealed an important contribution of cerebelo-cortical connectivity to cognitive dysfunction in PD (Palmer et al.). Motor symptom lateralization in PD also appeared to modulate cortico-striatal connectivity (Su et al.), which is known to influence the development and severity of cognitive and behavioral symptoms in PD.

Neuropsychiatric disturbances such as depression, apathy, hallucinations, impulse control disorders or irritability are also very common in movement disorders. They are especially frequent and severe in Lewy body dementia (DLB), which presents with the prototypical motor symptoms of PD but accompanied by severe and concomitant neuropsychiatric and cognitive dysfunction. Jaramillo-Jimenez et al. highlighted the important role of the amygdala in the development and trajectories of neuropsychiatric symptoms in DLB patients.

Subcortical deep brain stimulation in the subthalamic nucleus (STN) has been established as a highly-effective therapy for advanced Parkinson's disease. However, as Liu et al. showed, even though STN stimulation has shown clear benefits in terms of improving motor symptoms, they have also a detrimental impact on non-motor symptoms with significant impact in quality of life.

HD is a genetic neurological disorder in which patients experience progressive motor, cognitive, and neuropsychiatric alterations, resulting in a devastating loss of functional independence around the fourth decade of life. Even though the genetic alteration underlying HD is well-known, there is significant heterogeneity in the symptomatic trajectories across patients. For instance, it is currently unknown why some HD patients present with early and severe cognitive and neuropsychiatric disturbances, even in the absence of pronounced motor symptoms. Whereas, Zhang et al. reinforced the importance and heterogeneity of cognitive impairment in HD, Liu et al. showed that this heterogeneity is not explained by the inherent genetic burden, suggesting the involvement of additional pathological pathways whose characterization may reveal new therapeutic targets.

To conclude, through the use of neuroimaging, this Research Topic has contributed to advancing our understanding of cognitive and neuropsychiatric disturbances in Parkinson's disease and to disentangle cognitive heterogeneity in Huntington's disease. We thank the authors, the reviewers and the journal for their efforts leading to this collection.

## Author Contributions

All authors listed have made a substantial, direct, and intellectual contribution to the work and approved it for publication.

## Conflict of Interest

The authors declare that the research was conducted in the absence of any commercial or financial relationships that could be construed as a potential conflict of interest.

## Publisher's Note

All claims expressed in this article are solely those of the authors and do not necessarily represent those of their affiliated organizations, or those of the publisher, the editors and the reviewers. Any product that may be evaluated in this article, or claim that may be made by its manufacturer, is not guaranteed or endorsed by the publisher.

